# Lessons learned from Okinawa, Japan underscore the importance of clinical epidemiology in COVID-19 prevention and control

**DOI:** 10.1265/ehpm.25-00310

**Published:** 2026-02-05

**Authors:** Takuji Kishimoto, Daisuke Tasato, Yoshitaka Nagasawa, Akihiro Yamashiro, Hayashi Shokita

**Affiliations:** 1 Department of Health Screening, Okinawa North Medical Association Hospital, 1712-3 Nago City, Okinawa 905-8611, Japan; 2 Department of Respiratory and Infectious Diseases, Okinawa North Medical Association Hospital, 1712-3 Nago City, Okinawa 905-8611, Japan; 3 Department of Endocrinology, Metabolism and Dialysis, Okinawa North Medical Association Hospital, 1712-3 Nago City, Okinawa 905-8611, Japan; 4 Department of Gastroenterology, Okinawa North Medical Association Hospital, 1712-3 Nago City, Okinawa 905-8611, Japan

**Keywords:** COVID-19, Clinical epidemiology, Study design, Evaluation of testing, Evaluation of treatment, Policy decision-making

## Abstract

**Background:**

This letter highlights the importance of clinical epidemiology in COVID-19 prevention and control, based on experiences at a core hospital in northern Okinawa, Japan, and relevant literature.

**Methods:**

Using data from 5,097 COVID-19 patients, we conducted: (1) a descriptive cross-sectional study analyzing cases by person, time, and place; (2) an analytical cross-sectional study linking health checkup data to identify factors associated with severity (published in Environmental Health and Preventive Medicine); and (3) a cohort study of healthcare workers exploring determinants of post-vaccination antibody titers (published in Journal of Clinical Virology Plus).

**Results:**

The descriptive study showed cases rose from 70 in 2020 to 891 in 2021 and 3,995 in 2022, before declining to 141 in 2023. In the analytical cross-sectional study (n = 1,353), protective factors against severity included vaccination (odds ratio [OR] [2 doses vs. 0 or one doses]: 0.223, 95% confidence interval [CI] 0.114–0.436; OR [≥3 doses vs. 0 or one doses]: 0.090, 95% CI 0.035–0.229) and regular exercise (OR [“yes” vs. “no”]: 0.458, 95% CI 0.242–0.866). In the cohort study (n = 354), lower antibody titers (lowest quartile) were more likely among older adults (hazard ratio [HR] 5.82 for 40s vs. 20s, 95% CI 2.05–16.51; HR 9.96 for 60s vs. 20s, 95% CI 3.07–32.34) and drinking habits (HR 2.26 for “daily” vs. “never”, 95% CI 1.17–4.34).

**Conclusion:**

These findings, supported by related literature, demonstrate that clinical epidemiology played vital roles in monitoring infection trends, evaluating diagnostic and preventive measures, establishing treatment strategies, optimizing healthcare resources, and guiding policy. Its continued application will be essential for preparedness against future emerging infectious diseases.

## Graphical Abstract

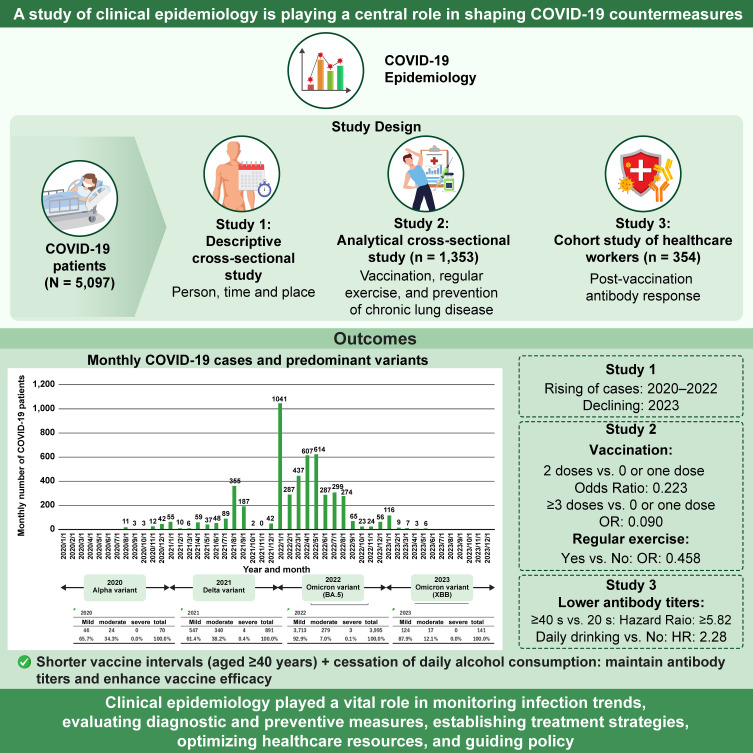

## 1. Background

Okinawa Prefecture has recorded the highest COVID-19 infection rate in Japan. Our hospital, a core facility in northern Okinawa, admitted its first patient on August 9, 2020, and treated 5,097 patients until May 8, 2023, when the disease was reclassified as “Category 5” under the Infectious Diseases Control Law. In the early phase, when preventive and therapeutic measures were not yet established, we adopted a hospital-wide approach guided by announcements from the Ministry of Health, Labor and Welfare, published literature, and our own clinical epidemiological analyses.

This experience emphasized the importance of clinical epidemiology, defined by the Japanese Society of Epidemiology as “the science of solving problems in clinical medicine using epidemiological methods” [1]. Table 1 presents a newly organized research design in clinical epidemiology, based on the previously published paper [2].

**Table 1:** Research designs in clinical epidemiology*

I. Descriptive			
	(1) Case report and case series		
		Introduction of symptoms and treatment history of target diseases	
	(2) Cross-sectional study		
		Describe the distribution of diseases and risk factors in a population at a specific point in time from the perspectives of “human attributes,” “time,” and “location.”	

II. Analytical			
	(1) Observational		
		① Cross-sectional study	
			Calculate the odds ratio of factors at a given point in time based on factors and disease
		② Case-control study	
			Efficiently examine risk factors for rare diseases
		③ Cohort study	
			Divide the group according to the presence or absence of risk factors and calculate the relative risk or the hazard ratio.
	(2) Experimental		
		① Interventional study	
			Assign interventions (such as treatment) and verify their effectiveness.

*: Newly organized research designs in clinical epidemiology based on the previously published paper [reference 2]

In this letter, we examine its role in COVID-19 prevention and control, drawing up studies conducted at our hospital and relevant literature.

## 2. Clinical epidemiological studies at our hospital

**Fig. 1:**
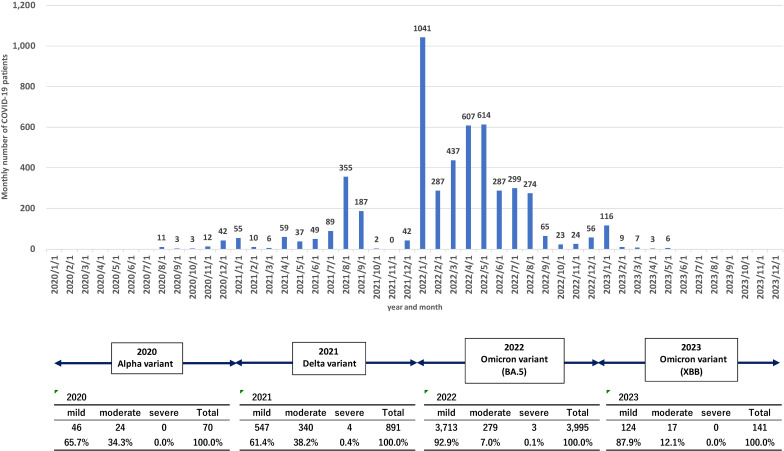
Monthly COVID-19 cases and predominant variants at our hospital (August 2020–May 2023) This figure shows the monthly number of COVID-19 patients (n = 5,097) at our hospital between August 9, 2020, and May 7, 2023. The bar graph illustrates monthly case counts, with values indicated above each bar. Major SARS-CoV-2 variants are displayed along the timeline at the bottom, showing the predominant strains during each period: Alpha in 2020, Delta in 2021, Omicron BA.5 in 2022, and Omicron XBB in 2023. The highest peak occurred in January 2022 (n = 1,041), corresponding to the early Omicron wave. Case numbers then gradually declined after mid-2022.

To evaluate the local infection situation, we conducted a descriptive cross-sectional study analyzing patient data by demographics, time, and location (Fig. 1, Table 2). Case numbers increased from 70 in 2020 to 891 in 2021 and 3,995 in 2022, then declined to 141 in 2023. The proportion of moderate-to-severe cases rose to 38.6% in 2021 but fell to 7.1% in 2022 and 12.1% in 2023, likely reflecting variant characteristics, vaccination coverage, improved treatment, and public health measures. Case number per 1,000 population was highest in Nago City (39.8) and lowest in Iejima Village (2.3), with higher rates on the main island than on remote islands, where population size and restricted mobility likely limited spread.

**Table 2:** Number of patients per 1,000 population in municipalities within the medical service area (2022)

**municipalities**	**location**	**Number of patients in 2022**	**population**	**Number of patients/1,000**
Nago City	main island	2,558	64,256	39.8
Nakijin Village	main island	327	8,894	36.8
Motobu Town	main island	478	13,016	36.7
Kunigami Village	main island	158	4,504	35.1
Higashi Village	main island	45	1,706	26.4
Ie Village	remote island	109	4,411	24.7
Ogimi Village	main island	69	3,025	22.8
Iheya Village	remote island	13	1205	10.8
Izena Village	remote island	3	1315	2.3

Total		3,760	102,332	36.7

To identify risk factors for disease severity, we performed an analytical cross-sectional study of 1,353 patients with prior health checkups, drawn from 4,899 diagnosed between August 2020 and December 2022. Linking medical records with checkup data, we found that vaccination, regular exercise, and prevention of chronic lung disease reduced the risk of severity. These research findings were published in a medical journal [3], and the main results are shown in Table 3.

**Table 3:** Main results of the published paper [reference 3]: Adjusted odds ratios* for each factor for exacerbation**

**factor**	**number of people**	adjusted odds ratio**	**95% confidence interval**			**statistical significance**

			**lower limit**		**upper limit**	
gender						
woman	658	1.000				
man	672	2.533	1.484	–	4.322	+

age						
10s + 20s + 30s	473	1.000				
40s	207	1.525	0.631	–	3.685	−
50s	266	4.858	2.319	–	10.177	+
60s	241	9.738	4.355	–	21.777	+
70s + 80s + 90s	139	8.327	3.224	–	21.507	+

year of consultation						
2020	24	1.000				
2021	109	1.849	0.617	–	5.541	−
2022	1,197	0.180	0.058	–	0.559	+

number of vaccinations						
0 times + once	305	1.000				
twice	626	0.223	0.114	–	0.436	+
3 times or more	399	0.090	0.035	–	0.229	+

chronic lung disease						
none	1,228	1.000				
yes	102	2.892	1.227	–	6.818	+

regular exercise^#^						
none	949	1.000				
yes	381	0.458	0.242	–	0.866	+

adjusted odds ratio*: Odds ratio using backward stepwise method for variables in binominal logistic regression analysis

exacerbation**: “mild” → “moderate or severe” of severity in patients with COVID-19

regular exercise^#^: exercising at least 2 days per week for at least 30 minutes each session at an intensity that causes a slight sweat for at least 1 year

Finally, a cohort study of 354 healthcare workers examined determinants of post-vaccination antibody titers. Antibody levels were measured five times over at least six months. Results indicated that shorter vaccine intervals among individuals aged ≥40 years and cessation of daily alcohol consumption may help maintain antibody titers and enhance vaccine efficacy. These research findings were published in a medical journal [4], and the main results are shown in Table 4.

**Table 4:** Main results of the published paper [reference 4]: Adjusted hazard ratios** of Lower 25%*

**factor**	**number of people**	**adjusted hazard ratio^‡^**	**95% confidence interval**			**statistical significance**
age						
20s	66	1.00				
30s	88	2.59	0.85	–	7.92	−
40s	99	5.82	2.05	–	16.51	+
50s	77	7.12	2.46	–	20.63	+
60s	24	9.96	3.07	–	32.34	+

frequency of drinking						
never	87	1.00				
sometimes	215	1.58	0.88	–	2.84	−
daily	52	2.26	1.17	–	4.34	+

Adjusted hazard ratios**: Adjusted hazard ratios of Lower 25% for lifestyle factors using Cox regression model

adjusted hazard ratio^‡^: using backward stepwise method for Cox regression model

Abbreviation: Lower 25%*: Antibody titer less than the 25th percentile on 6 months after the second vaccination

Together, these studies demonstrate the utility of clinical epidemiology in clarifying infection dynamics, identifying risk and protective factors, and informing preventive and vaccination strategies in COVID-19 management.

## 3. Broader importance of clinical epidemiology in COVID-19 prevention and control

### (1) The importance and challenges of clinical epidemiology in COVID-19 prevention and control: lessons learned from the various activities at our hospital

From the initial emergence of patients in 2020 until the Ministry of Health, Labour and Welfare published treatment guidelines, treatment methods remained unclear. The early morning (40-minute) resident seminars led by the vice president (co-author Yoshitaka Nagasawa) featured presentations on approximately five recent papers from international medical journals each session. These presentations formed the foundation for developing our hospital’s treatment guidelines. Until the pandemic subsided, these resident seminars served as a vital opportunity to share the latest findings on COVID-19. The referenced papers were primarily clinical epidemiological studies. It was highly beneficial that many international medical journals recognized the importance of publishing papers during the coronavirus pandemic and made COVID-19-related papers freely available. By the time the pandemic subsided, the vice president had conducted 107 resident seminars focused on COVID-19.

At the suggestion of the Head of the Respiratory and Infectious Diseases Department (co-author Daisuke Tasato), a team comprising a physician, nurses, and administrative staff from the Department of Health Screening visited nursing care facilities, including special nursing homes for the elderly within Nago City where our hospital is located, and administered vaccinations to approximately 500 residents. The necessity of this initiative was supported not only by the intuition of clinicians on the frontlines (who witnessed many elderly patients deteriorating to moderate or severe illness and rapidly occupying hospital beds) but also by data from a descriptive cross-sectional study conducted at our hospital. Many facility residents lack the means to travel to vaccination sites operated by local governments and are missing opportunities to receive the vaccine. This situation persists and represents a major challenge going forward.

Okinawa Prefecture had the highest infection rate per capita. Contributing factors include its vibrant tourism industry attracting many visitors from outside the prefecture and the presence of U.S. military bases, which bring in a large number of soldiers from the United States. The Omicron variant, considered highly transmissible but less virulent, is believed to have originated at these U.S. military bases. The United States is one of the countries hardest hit globally in terms of both the number of infections and deaths. The U.S. military bases within the prefecture belong to the U.S. state of California, and U.S. military personnel can freely enter and exit Okinawa bases. While adequate quarantine measures were initially lacking, the situation appears to have improved following strong requests from the prefecture. Some local medical institutions refused to accept COVID-19 patients. During the peak of the Omicron variant spread in 2023, patient numbers surged dramatically, leaving physicians in Department of Respiratory and Infectious Disease facing overwhelming workloads and nearing burnout. The entire hospital participated in countermeasures across departmental boundaries, treating mild cases and conducting daily telephone health checks for patients under home quarantine. Descriptive cross-sectional studies targeting hospitalized patients are highly effective but require on-site clinical epidemiologists and personnel to create patient information databases. At our hospital, we established the COVID-19 Task Force at the Okinawa Northern Medical Association Hospital. The nursing department entered patient information into Excel to build the database. A physician from the Department of Health Screening handled the descriptive cross-sectional study analysis. Very few core hospitals in the prefecture were able to establish such a system, which is considered a major challenge for the future.

Around the time vaccinations became widely available in 2022, evidence demonstrating their effectiveness began to emerge gradually. However, vaccination faced criticism lacking scientific basis. Amidst this, a cross-sectional analysis study conducted at our hospital clearly demonstrated with concrete data that vaccination prevents worsening of disease severity. This finding provided significant impetus for vaccination implementation. To communicate these results to the people of Okinawa, we posted the English paper and a Japanese summary on our hospital website and contributed an opinion piece to a local Okinawan newspaper. Subsequently, the Mayor of Nago requested the implementation of a special vaccination program for adult citizens. We then suspended our regular health checkup services for two months, allowing the Department of Health Screening to vaccinate approximately 20,000 citizens.

Vaccinations were first administered to healthcare workers before the public. We conducted a cohort study measuring antibody titers among our hospital’s healthcare workers to evaluate vaccine efficacy. Results indicated that shorter vaccine intervals among individuals aged ≥40 years and cessation of daily alcohol consumption may help maintain antibody titers and enhance vaccine efficacy. These findings were reported to the public through the posting of the English paper and Japanese summary on our hospital website, as well as through the opinion piece in the Okinawan local newspaper.

For the reasons stated above, the clinical epidemiology conducted at our hospital played an extremely important role in COVID-19 countermeasures. We also examined issues related to establishing an environment suitable for implementing clinical epidemiology.

### (2) The importance of clinical epidemiology learned from our hospital’s clinical epidemiology research and the latest international research paper

Based on the clinical epidemiological studies conducted at our hospital and the relevant literature, we outline the diverse roles of clinical epidemiology in COVID-19 countermeasures (Table 5).

**Table 5:** Importance of clinical epidemiology in COVID-19 prevention and control

**Territory**	**Role of clinical epidemiology**
Understanding epidemiological indicators and assessing risks	Evaluation of infection, severity, mortality rates Identification of risk factors
Evaluation of the effectiveness of inspection and diagnostic methods	Evaluation of sensitivity and specificity of PCR tests and antigen tests Effectiveness of screening asymptomatic individuals
Verification of the effectiveness of preventive measures (vaccines, masks, behavioral changes)	Proving the efficacy and safety of vaccines Evaluation of non-pharmacological interventions such as masks, hand washing, and ventilation
Evaluation of the efficacy and safety of treatment methods	Verification of the efficacy of therapeutic drugs Understanding the reality of long-term sequelae (Long COVID) and countermeasures
Optimal allocation of medical resources	Development of a model for predicting severe cases Establishment of triage criteria
Contributions to health policy and public health measures	Evidence-based decision support Support for risk communication

Clinical epidemiology was used to monitor infection dynamics and identify high-risk groups [3, 5]. Epidemiological indicators such as incidence, severity, and mortality clarified risks in older adults and individuals with underlying conditions (e.g., diabetes, obesity), providing evidence for prioritizing protective measures.

It has also been applied to evaluate testing and diagnostic methods [6]. The accuracy and reliability of these tools were assessed, leading to appropriate guidelines and forming the basis for cluster control and contact tracing strategies.

Preventive measures have been validated through epidemiological research. Vaccines, mask use, and behavioral changes were shown to reduce severe illness and death, supporting vaccination policy. Non-pharmaceutical interventions such as mask-wearing, hand hygiene, and ventilation were similarly evaluated, strengthening policy decisions [7].

Randomized controlled trials and cohort studies assessed the efficacy and safety of treatments, while observational studies described the symptoms, prevalence, and risk factors of long-term sequelae (Long COVID) [8].

In optimizing healthcare resources, clinical epidemiology contributed to predictive models identifying patients likely to require intensive care or ventilation. These models provided evidence for triage criteria and guided treatment and hospitalization priorities [9].

Finally, clinical epidemiology informed public health policy and risk communication. Evidence supported interventions such as movement restrictions, emergency declarations, and vaccine prioritization [10]. Accurate, evidence-based risk communication fostered behavioral change and public cooperation.

Collectively, these findings highlight the central role of clinical epidemiology in shaping COVID-19 countermeasures. Its continued application will be vital for mitigating future outbreaks and responding to emerging infectious diseases.
